# Variation in the Adult Sex Ratio and Morphological Traits of *Cardisoma guanhumi* (Latreille, 1828) in Contrasting Habitats in the Southwest of the Gulf of Mexico

**DOI:** 10.1002/ece3.71710

**Published:** 2025-07-09

**Authors:** Jared Leyva‐Hernández, Martha L. Baena, Ivette Alicia Chamorro‐Florescano, Israel Huesca‐Domínguez

**Affiliations:** ^1^ Facultad de Ciencias Biológicas y Agropecuarias de Tuxpan, Doctorado en Ciencias Marinas y Costeras Universidad Veracruzana Xalapa Veracruz México; ^2^ Instituto de Investigaciones Biológicas Universidad Veracruzana Xalapa Veracruz México

## Abstract

The habitat and its environmental conditions, when optimal, influence the reproduction and survival of organisms, since they can have an impact on demographic parameters such as adult sex ratio (ASR), density, and functional traits (morphological, physiological). Few studies evaluate these factors as a whole in contrasting habitats (such as mangroves and pastures), especially in crab species such as *Cardisoma guanhumi* . We hypothesized that mangroves would be a favorable habitat for *C. guanhumi* at a local and regional scale because the environmental conditions (lower temperature, soft and clayey substrate) would have a positive effect on its population dynamics and morphological traits. In order to test this hypothesis, we selected 12 sites on the coasts of the Gulf of Mexico. We found an effect of habitat on ASR, which was male‐biased in the mangrove and female‐biased in the pasture at a local (by site) and regional scale. Furthermore, crab density was higher in the mangrove and decreased in both habitats as soil hardness increased. In addition, females were heavier and larger (quela, carapace, ventral plate) in the mangrove, and these traits were positively related to burrow temperature in this habitat. Our results support the hypothesis that mangroves are the optimal environment for the development of *C. guanhumi* . However, although this threatened species has been able to adapt to modified habitats such as pastures, its vulnerability is increased by the surrounding environmental conditions, predation, and commercial exploitation, and the viability of its populations may be affected if it persists in these modified environments.

## Graphical abstract

At a regional scale, we found a bias toward one sex according to the habitat and differences in weight and size between females and males.

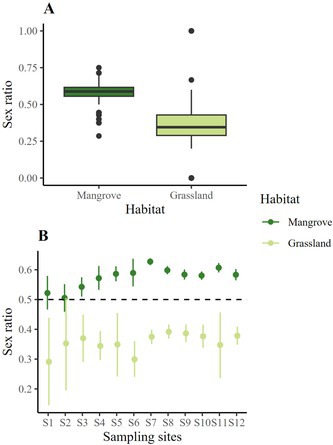

## 1 Introduction

The habitat is a factor that can influence the demographic parameters of populations as well as the adult sex ratio (ASR) (Amos et al. 2013; Fromhage and Jennions 2016; Kappeler et al. 2023) and density of individuals (Julliard 2000; Reichard et al. 2014; Barretto et al. 2022). Although insufficiently explored, the habitat may also influence the morphological and physiological traits of a population (Yates et al. 2014; Kappeler et al. 2023). Moreover, populations can interact with the habitat in different ways as a response to its environmental conditions (Xu et al. 2016; Alonso‐Fernández et al. 2017; Wittmann et al. 2023). Optimal environmental conditions in the habitat (resource availability and favorable microclimate) are known to play a role in the reproduction and survival of individuals, which in turn affect the ASR (Julliard 2000; Reichard et al. 2014; Jennions and Fromhage 2017; Edmands 2021; Bókony et al. 2024; Verma et al. 2024). However, the relationship between population spatial dynamics and habitat properties is complex and scarcely supported by field data (Wittmann et al. 2023; Marrero et al. 2025).

The ASR includes individuals in a population that have reached sexual maturity whether they are sexually active or not (Hardy 2002; Fromhage and Jennions 2016; Ancona et al. 2017; Carmona‐Isunza et al. 2017). Adult sex ratio and density are parameters that regulate the structure of a population and play a crucial role in population dynamics, since they influence reproductive success (competition for mates and resources) and fecundity and mortality rates, among others (Clutton‐Brock 1991; Hardy 2002; Julliard 2000; Steifetten and Dale 2012; Ancona et al. 2017; Jennions and Fromhage 2017; Végvári et al. 2018; Schacht et al. 2022; Kappeler et al. 2023). Fisher's classic work (1930) predicts that the sex ratio is at equilibrium 1:1 when the costs of producing offspring are similar. However, under natural conditions, the ASR tends to be biased towards one sex due to the selective pressure of different ecological factors (environmental conditions, habitat loss, resource availability). Therefore, the ASR is expected to be biased towards the sex with a better ability to exploit the environmental conditions of the habitat (Julliard 2000; Steifetten and Dale 2012; Végvári et al. 2018; Barretto et al. 2022; Kappeler et al. 2023). Additionally, male‐biased ASRs and high population densities can be found in optimal or good‐quality habitats, where predation mortality is low (Julliard 2000; Kappeler et al. 2023). Under these conditions, intense sexual selection can be observed, that is, high reproductive skewness, intense competition, and a high risk of exclusion from mating among males (Emlen and Oring 1977; Kokko and Rankin 2006; Jennions and Fromhage 2017).

Some studies have shown a relationship between temperature and functional traits. In some species, temperature modulates anatomical specialization and biological processes (physiological, developmental) in relation to the “size‐temperature rule” which occurs in most ectotherms (Verberk et al. 2021). For example, when reared in warmer conditions, a smaller body size at maturity is observed in marine populations of *Littoraria angulifera* (Lamarck, 1822), where body size decreased in anthropogenic habitats (urban intertidal artificial structures) characterized by higher temperatures than in adjacent natural habitats (Ramos et al. 2021). In *L. angulifera* , decreasing developmental time and reaching maturity at a smaller size seems to favor fertility, and therefore, the adult sex ratio would be higher than that suggested by the size frequency distribution (Ramos et al. 2021).

Temperature is also a key factor in shaping the geographic distribution of species and the density and ASR variation of populations, especially in species that can establish in different types of habitats (Rohde 1992; Edmands 2021; Verma et al. 2024). In brachyuran crabs such as *Ucides occidentalis*, *Cardisoma crassum*, and *Cardisoma guanhumi* , environmental conditions such as temperature (Burgos and Garófalo 2018; Lombardo and Rojas 2022; Mota et al. 2023), vegetation cover, and/or soil hardness (Capistrán‐Barradas et al. 2003; Carmona‐Suárez and Guerra‐Castro 2018) have been found to primarily influence the density of individuals.

Among decapods, *C. guanhumi* is known to be one of the best terrestrial colonizers. Terrestrial females have been found to migrate periodically to the sea in order to spawn, thus their larvae are strictly marine pelagic (Gifford 1962). Hence, the early developmental stages of this species, and several other semi‐terrestrial crabs, are found within a narrow strip of the coast (Hartnoll et al. 2014). As a semi‐terrestrial species, adults of *C. guanhumi* can expand their habitable area and build their burrows further away from the seashore. *Cardisoma guanhumi* burrows have been recorded at distances from 300 m to 5 km from the sea (Carmona‐Suárez and Guerra‐Castro 2012; Santos et al. 2016; Novais et al. 2021). Thus, this crab species can establish itself in different types of habitats, from dense mangrove forests, with hydroperiods and humid soils, to transformed environments such as sparsely vegetated pastures, with higher temperatures than mangroves and superficially arid and compacted soils, resulting from human activities (Rodríguez‐Fourquet and Sabat 2009; Giménez et al. 2015; Mendes and Cruz 2017). It has also been shown that, although pastures are habitats that represent a loss of hydrological connectivity with the sea, they provide a stable substrate for *C. guanhumi* to build its burrows, since the burrows have a connection with groundwater from the water table, which allows the crabs to survive in these disturbed habitats (Arroyave‐Rincón et al. 2014). *Cardisoma guanhumi* is a crab species that is distributed from the south of Florida to Brazil, including the Caribbean islands and West Africa (Govender and Rodríguez‐Fourquet 2008). In Mexico, *C. guanhumi* is distributed in the Caribbean and much of the Gulf of Mexico (Amaral et al. 2015), where ≈10% of the mangrove vegetation cover has been replaced by pastures for agricultural activities, aquaculture, livestock farming, urbanization, and coastal development (reviewed in Osland et al. 2022). Pastures are environments with high temperatures, very compact soils, and vegetation cover with little shade (Hernández‐Melchor et al. 2016).

In a comparison between mangroves and pastures, Govender and Rodríguez‐Fourquet (2008) suggested that *C. guanhumi* individuals are more abundant in mangroves due to minimal fluctuations in soil surface temperature and easy access to groundwater. However, there is a debate about the effect of the habitat on some demographic parameters. Some studies have found that habitat modifications due to land‐use change can result in more abundant and larger individuals in mangroves compared to pastures (Arroyave‐Rincón et al. 2014; Carmona‐Suárez and Guerra‐Castro 2018; Quiñones‐Llópiz et al. 2021). Other studies have not found an effect of habitat but have found an effect of physical factors (shade, plant litter, and soil substrate) or landscape scale on the abundance and size of *C. guanhumi* (Novais et al. 2021; Riascos et al. 2024). On the other hand, a systematic review of 25 studies covering the latitudinal distribution of this species shows that, in areas near the southern limit of its distribution, crab populations are more abundant and larger in urban areas and farmlands than in natural habitats (Riascos et al. 2024). Thus, it is not known how habitat type and environmental conditions can influence demographic parameters such as ASR, density, and morphological traits in species like *Cardisoma guanhumi* (Latreille, 1825). This approach allowed us to analyze the response of *C. guanhumi* to these factors at both the local and regional scale on the coasts of Tamaulipas and Veracruz in the Gulf of Mexico.

Considering the above, we hypothesized that mangroves would be a favorable habitat for *C. guanhumi* at a local and regional scale due to its environmental conditions (lower temperature, soft and clayey substrate) having a positive effect on its population dynamics and morphological traits. We predicted that *C. guanhumi* populations in mangroves would show a higher mean body condition (size, weight), higher density, and a biased ASR compared to those found in pastures. The transformation of a natural habitat (mangroves) into pastures can have a potential negative effect on the availability of suitable sites for reproduction (burrows) and food resources necessary for survival.

## 2 Materials and Methods

### 2.1 Study Area

**FIGURE 1:**
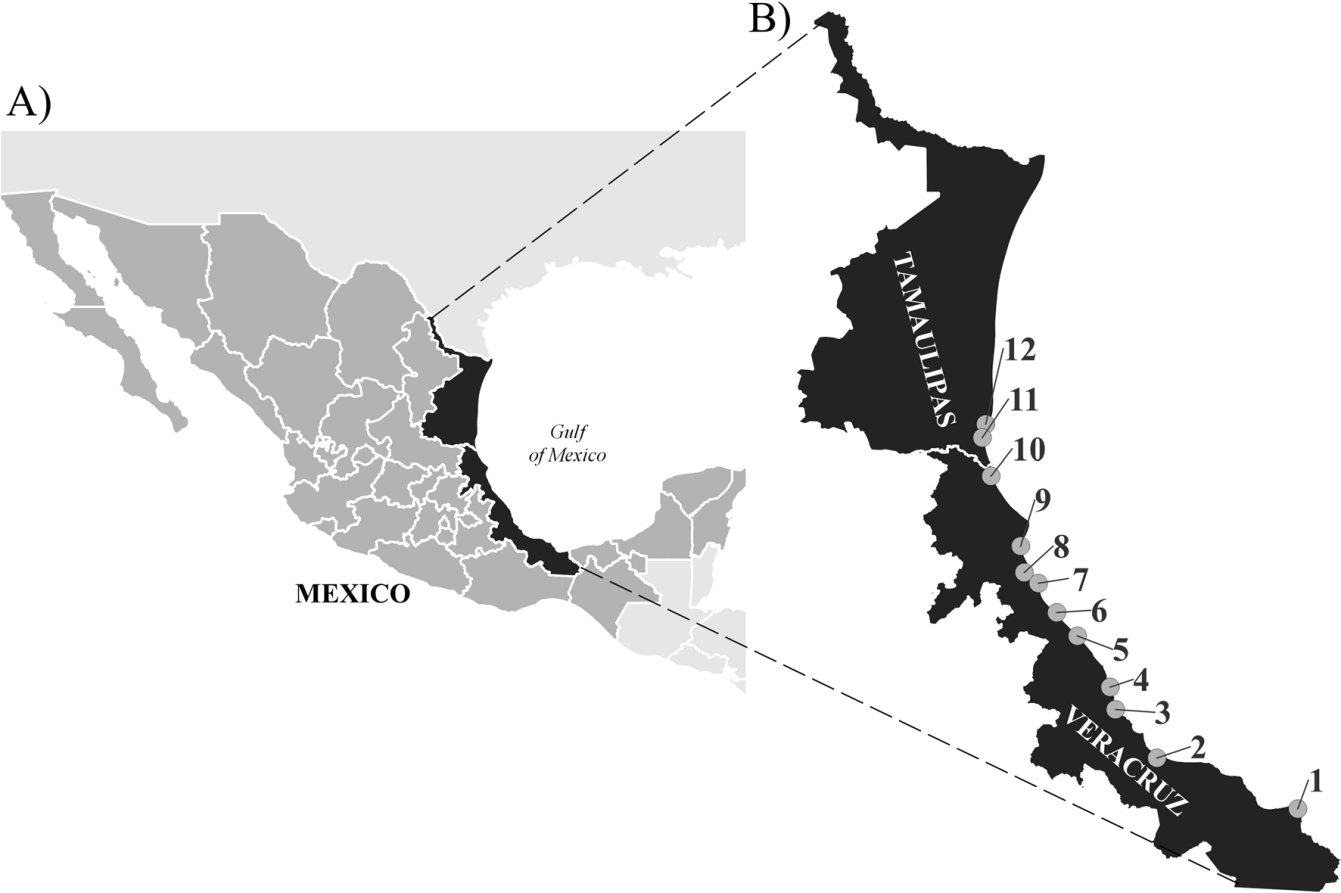
Distribution of the sampling sites of *Cardisoma guanhumi* on the coasts of Tamaulipas and Veracruz (Gulf of Mexico: 18.14°–23.78° Latitude N; 94.14°–97.90° Longitude W). (1) Agua Dulce. (2) Alvarado. (3) La Antigua. (4) La Mancha. (5) Nautla. (6) Tecolutla. (7) Chile Frio. (8) Tuxpan. (9) Tamiahua. (10) Tampico Alto. (11) Aquiles Serdán. (12) El Morón. Sites (1 and 2)—Tamaulipas. Sites (3–12)—Veracruz.

The present study was conducted at a regional scale on the coasts of Tamaulipas and Veracruz in the Gulf of Mexico (18.14°–23.78° N; 94.14°–97.90° W). Twelve sampling sites where the crabs were not harvested (to rule out anthropogenic effects) were established: two in Tamaulipas and 10 in Veracruz (Figure 1). Since *C. guanhumi* is able to occupy different habitats (as long as it has access to the water table) (Rodríguez‐Fourquet and Sabat 2009; Mendes and Cruz 2017; Novais et al. 2021), we selected two types of habitats at a local and regional scale in the Gulf of Mexico: mangrove forests as a natural habitat and pastures as a disturbed habitat. The pastures in this study are characterized by the presence of grasses and some herbaceous plants and the absence of species such as red mangrove ( *Rhizophora mangle* ), white mangrove ( *Laguncularia racemosa* ), and black mangrove ( *Avicennia germinans* ), which are species found in mangrove habitats in Mexico (Portillo and Ezcurra 2002). Mangroves and pastures were selected in each site and sampled during September and October of 2021 and 2022, which are the months when adults are present (Carmona‐Suárez and Guerra‐Castro 2018; Quiñones‐Llópiz et al. 2021). Despite sampling the northern sites in one year and the southern sites in the following year, the complete statistical model did not show a significant effect of year on the study parameters. Furthermore, each sampling site was only surveyed in a single year due to time constraints, as the northern sites are 700 km away from the southern sites on the coast of the Gulf of Mexico.

### 2.2 Sampling Design

In each selected habitat (mangrove and pasture) per sampling site, we established five 50‐m‐long transects separated by a distance of *≈*10 m from each other. To locate the burrows of *C. guanhumi* , we established three 5 × 5 m quadrats in each transect, resulting in a total of 15 quadrats/habitat. The quadrats in the mangrove were placed at a distance of ≈30 m from the nearest body of water, avoiding flooded areas. The same design was used in each of the 12 sites. A total of 180 quadrats were established in mangrove habitats and 180 in pasture habitats along the coasts of Tamaulipas and Veracruz. We recorded the total number of crabs found inside their burrows in each quadrat in order to quantify the abundance and density of individuals per site and habitat. For this, we extracted the individuals from their burrows using a 2.5‐m‐long flexible crab hook, ensuring not to damage the morphological structures.

We only recorded adult individuals, which were determined by a carapace width equal to or greater than 58.5 mm, since color is not always linked to body size (Arroyave‐Rincón et al. 2014). The following parameters were recorded for each captured individual: sex, carapace width and length, width, length, and thickness of the largest quela, maximum width of the ventral plate, and body weight. We used an AutoTEC digital caliper to measure the structures (0.01 precision) and a TORREY LAB‐500 portable digital balance (0.1 g precision and 500 g capacity) to weigh each individual. All the data were obtained in situ, and the surveyed and measured individuals were released alive in their respective habitat (mangrove or pasture). We also recorded the environmental temperature and the temperature inside the burrows using a digital thermometer. Burrow temperature was obtained by pointing a digital laser infrared thermometer (GM 550, 10°C–55°C) at the deepest accessible part of the burrow. Soil compaction was measured in both mangroves and pastures using a Siless penetrometer.

### 2.3 Statistical Analysis

Crab density was calculated as the number of individuals per quadrat (25 m^2^). Adult sex ratio was estimated as: total number of males/(males + females). This estimator is asymmetric around 0.5, where a value of 0 indicates a female‐biased ASR and a value of 1 a male‐biased ASR (Hardy 2002; Ancona et al. 2017).

We used a generalized linear mixed model (GLMM) to analyze the effect of the predictor variables of habitat (mangrove and pasture) and the environmental conditions (soil hardness, ambient temperature, burrow temperature) on ASR (response variable with a binomial distribution and logit link function). The same predictor variables were used for the response variable of density in a GLMM with a Gamma distribution and log link function.

Spatial variation of the ASR of *C. guahnumi* in 12 sampling sites on the coasts of Tamaulipas and Veracruz (Gulf of Mexico) was analyzed by a non‐parametric bootstrapping procedure (10,000) to determine the 95% asymmetric confidence intervals (Davison and Hinkley 1997) using the R package *boot* (Canty and Ripley 2017).

To determine the relationship and reduce the dimensionality among the morphological variables, we performed a principal component analysis (PCA). We then used linear mixed models (LMMs) to evaluate whether the morphological traits of *C. guanhumi* (weight, carapace width, quela thickness, and ventral plate width) varied with sex (females and males), habitat, and environmental conditions (soil hardness, environmental temperature, and burrow temperature). For all models, sampling site was considered a random factor and habitat and sex as fixed factors. We used the R package *lme4* (Bates et al. 2015) for the mixed models, and all analyses and data visualization were performed in R version 4.4.1 (R Core Team 2024).

## 3 Results

We sampled a total of 3618 crabs and found 1042 females (28.8%) and 1459 males (40.32%) in the mangrove and 709 females (19.6%) and 408 males (11.28%) in the pasture. The mean density (±SD) per quadrant was 13.89 ± 5.43 individuals in the mangrove and 6.41 ± 3.24 individuals in the pasture.

### 3.1 Adult Sex Ratio (ASR) and Crab Density

**FIGURE 2:**
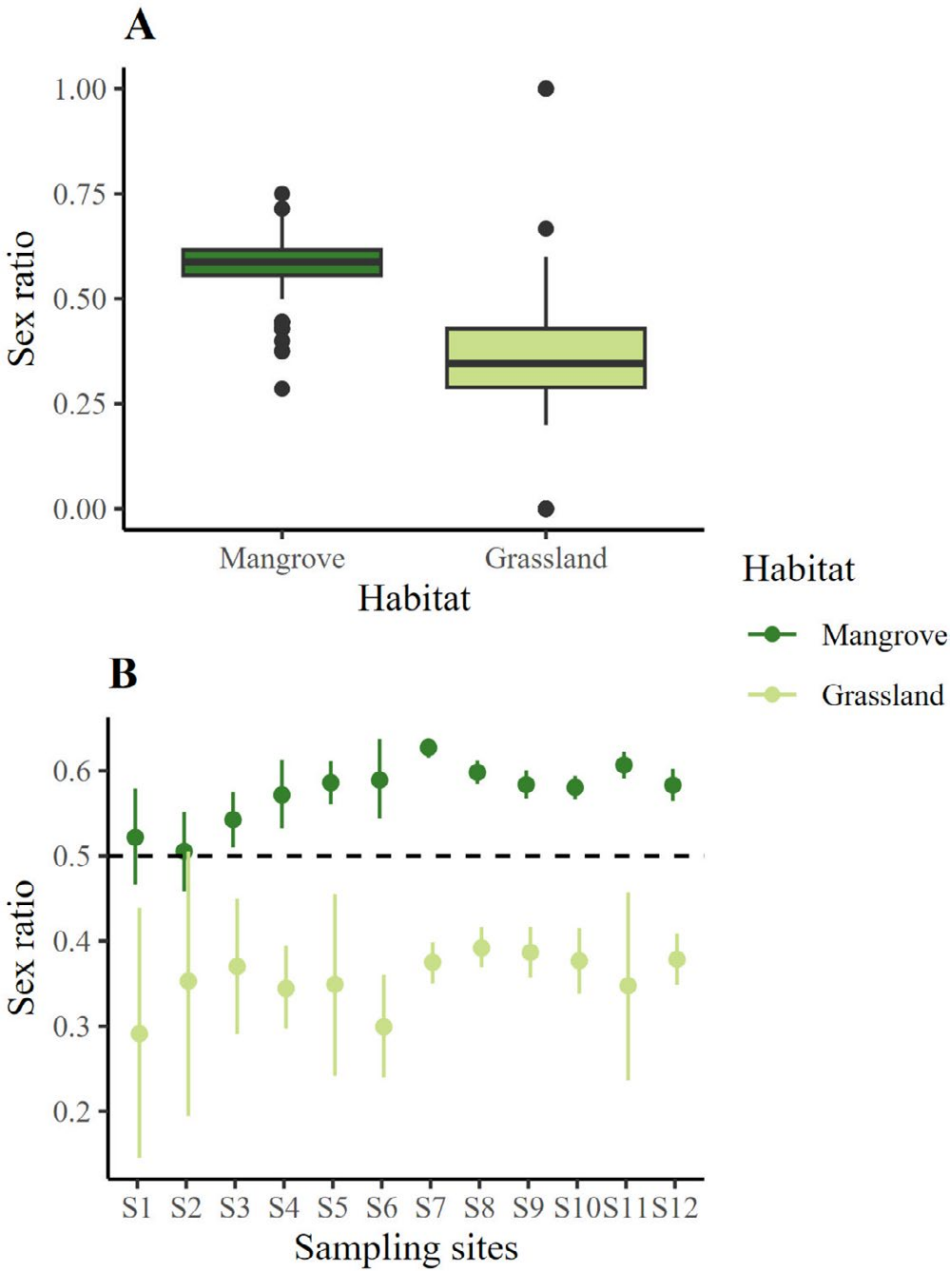
Adult sex ratio (ASR) of *Cardisoma guanhumi* as a function of (A) habitat (local scale) and (B) sampling site (regional scale) on the coasts of Tamaulipas and Veracruz (Gulf of Mexico).

Of the environmental variables analyzed as predictors of ASR, the GLMM only showed a significant effect of habitat (*χ* ^2^ = 7.14, df = 1, *p* < 0.001, Table 1). The ASR was male‐biased in the mangrove and female‐biased in the pasture (Figure 2A). This difference in ASR between habitats (local scale) was also found among the 12 sites sampled along the coasts of Tamaulipas and Veracruz in the Gulf of Mexico, since the confidence intervals per habitat did not overlap, which indicates that the ASR is consistently biased towards each sex: male‐biased in the mangrove and female‐biased in the pasture (Figure 2B).

**TABLE 1:** Generalized linear mixed model (GLMM) of predictor variables (fixed effects) of the adult sex ratio (ASR) of *Cardisoma guanhumi* on the coasts of Tamaulipas and Veracruz (Gulf of Mexico).

Fixed effects	*χ* ^2^	df	*p*
Habitat	7.14	1	**< 0.001**
Soil hardness	1.19	1	0.28
Environmental temperature	0.0035	1	0.95
Burrow temperature	0.51	1	0.47
Density	0.018	1	0.89

*Note:* Significant *p* values are highlighted in bold.

**FIGURE 3:**
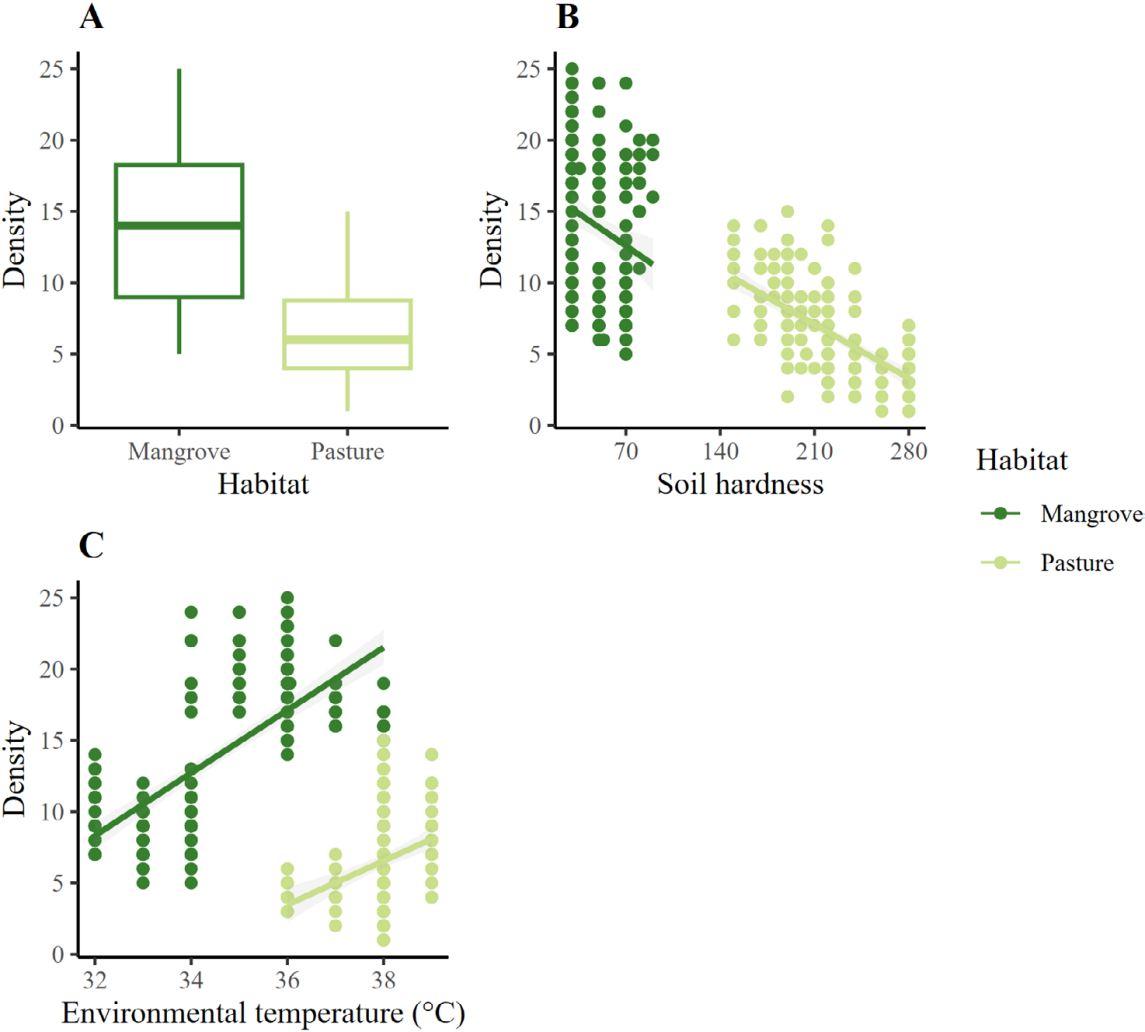
Variation in the density of *Cardisoma guanhumi* according to (A) habitat type, (B) soil hardness, and (C) environmental temperature.

The GLMM showed a significant effect of habitat (*χ* ^2^ = 52.749, df = 1, *p* < 0.001), soil hardness (*χ* ^2^ = 7.592, df = 1, *p* < 0.001), and environmental temperature (*χ* ^2^ = 6.327, df = 1, *p* < 0.001) on density. Crab density was higher in the mangrove than in the pasture (Figure 3A) and decreased as soil hardness increased in both habitats (Figure 3B). In contrast, there was a positive relationship between environmental temperature and crab density, particularly in the mangrove. Temperature varied more in the mangrove than in the pasture (Figure 3C).

### 3.2 Influence of the Habitat on the Morphological Traits of *C. guanhumi* on the Coasts of Tamaulipas and Veracruz (Gulf of Mexico)

The PCA represented 92.88% of the total variation of the data. The first component represented 82.73% of the total variance, where carapace width and length and quela width were the best represented. The second component represented 10.15% of the total variation, where ventral plate width was the best represented variable, while the carapace and quela measurements showed an inverse relationship (Table S1). Carapace width (mean ± SD) measured 7.06 ± 1.01 in the mangrove and 5.5 ± 0.69 in the pasture for females and 5.28 ± 0.8 in the mangrove and 6.93 ± 0.81 in the pasture for males (Table S2).

**FIGURE 4:**
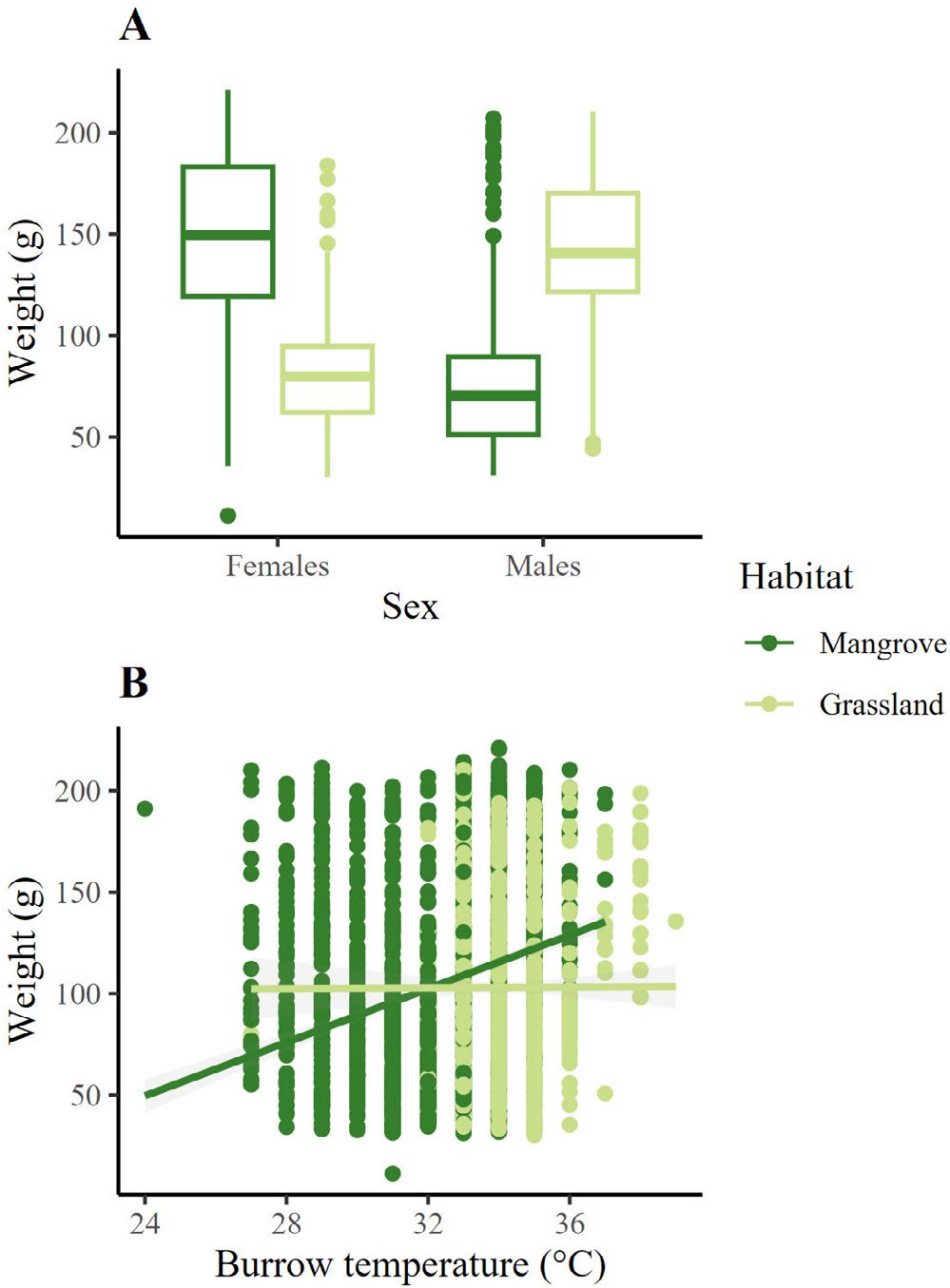
(A) Influence of sex and habitat on the weight of *Cardisoma guanhumi* , where females were heavier in the mangrove. (B) Relationship predicted by the LMM between burrow temperature and the weight of *C. guanhumi* on the coasts of Tamaulipas and Veracruz (Gulf of Mexico).

The minimal model from the LMM showed that sex (*χ* ^2^ = 693.162, df = 1, *p* < 0.001), burrow temperature (*χ* ^2^ = 33.094, df = 1, *p* < 0.001) and the interaction habitat:sex (*χ* ^2^ = 3524.938, df = 1, *p* < 0.001) had a significant effect on the weight of *C. guanhumi* (Table 2). The interaction shows that females are heavier in the mangrove, while males are heavier in the pasture (Figure 4A). We also found that weight increased with burrow temperature in the mangrove (Figure 4B).

**TABLE 2:** Linear mixed model (LMM) of predictor variables of the morphological traits of *Cardisoma guanhumi* on the coasts of Tamaulipas and Veracruz (Gulf of Mexico).

Predictor variables	Body weight			Chela thickness		
Fixed factors	*χ* ^2^	df	*p*	*χ* ^2^	df	*p*
Habitat	1.148	1	0.2839	1.67	1	0.20
Sex	693.162	1	**< 0.001**	151.76	1	**< 0.001**
Soil hardness	0.577	1	0.4472	0.10	1	0.76
Environmental temperature	0.001	1	0.9703	0.00	1	1.0
Burrow temperature	33.094	1	**< 0.001**	2.16	1	0.14
Habitat:sex	3524.938	1	**< 0.001**	569.71	1	**< 0.001**

*Note:* Significant values are highlighted in bold.

**FIGURE 5:**
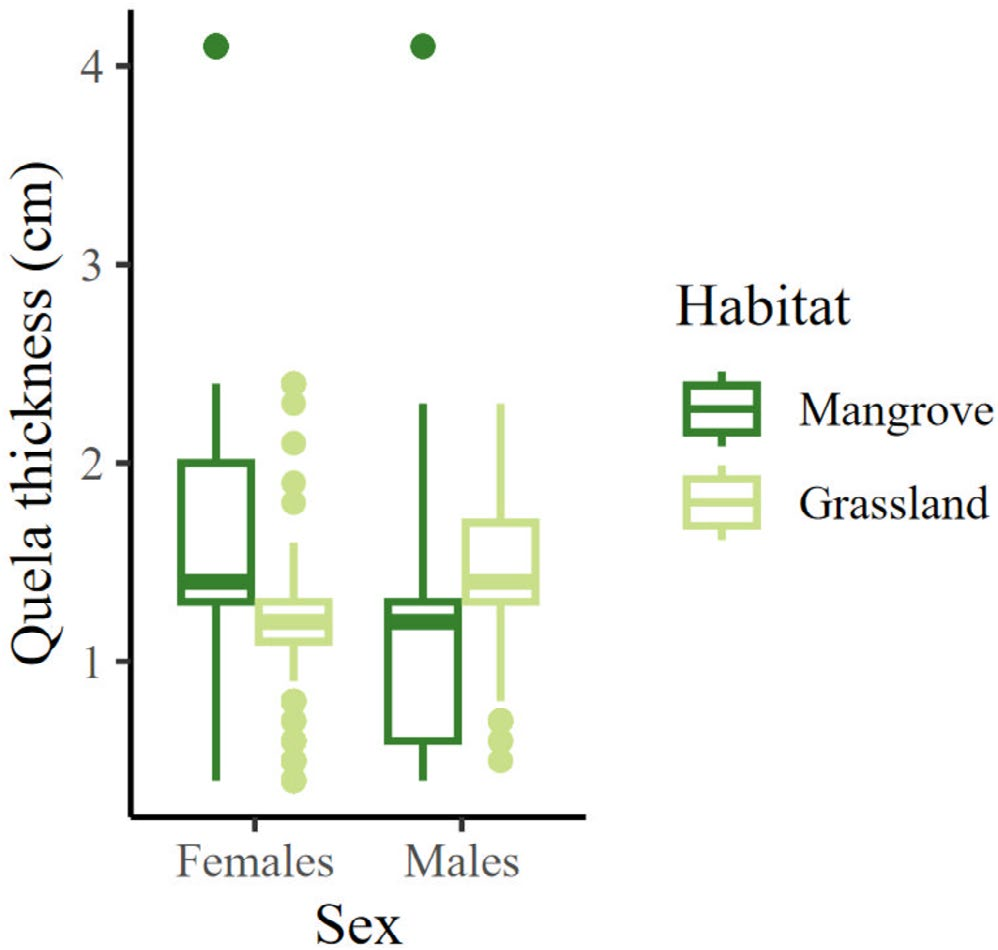
Chela size of females and males in contrasting habitats. Mean and extreme values are represented.

In the case of quela thickness, only sex and the interaction sex:habitat showed a significant effect (Table 2). Females had thicker quelea in the pasture than in the mangrove, and males showed the opposite pattern (Figure 5; Table S2).

**FIGURE 6:**
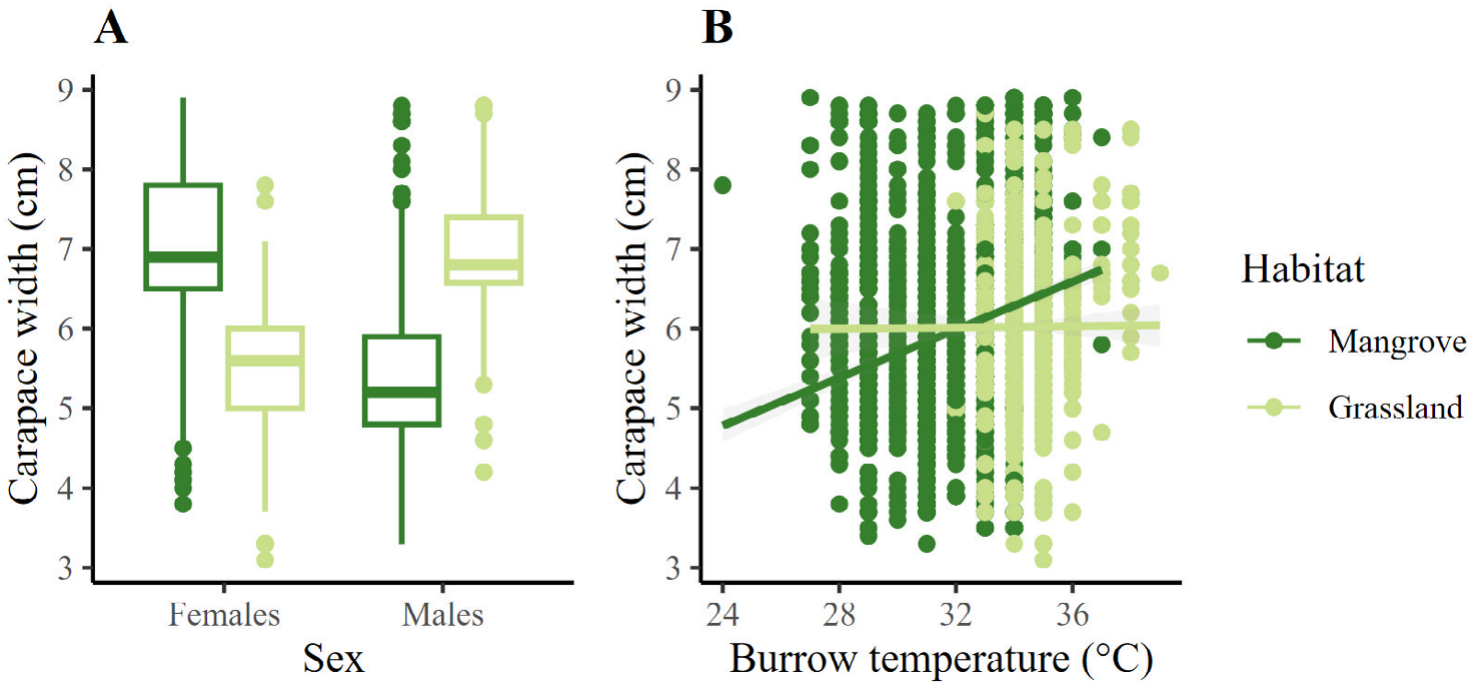
(A) Carapace size of females and males in contrasting habitats. Mean and extreme values are represented. (B) Relationship predicted by the LMM between carapace size and burrow temperature, where carapace size increased with burrow temperature in the mangrove.

Sex, burrow temperature, and the interaction habitat:sex had a significant effect on carapace width (Table 3). The carapace of females was wider in the mangrove, while the carapace of males was wider in the pasture (Figure 6A). Carapace width increased with burrow temperature in the mangrove (Figure 6B).

**TABLE 3:** Linear mixed model (LMM) of predictor variables of the morphological traits of *Cardisoma guanhumi* on the coasts of Tamaulipas and Veracruz (Gulf of Mexico).

Predictor variables	Carapace width			Ventral plate width		
Fixed factor	*χ* ^2^	df	*p*	*χ* ^2^	df	*p*
Habitat	1.26	1	0.26	12.00	1	**< 0.001**
Sex	577.33	1	**< 0.001**	16329.02	1	**< 0.001**
Soil hardness	0.37	1	0.54	0.018	1	0.93
Environmental temperature	0.00	1	1.00	0.05	1	0.82
Burrow temperature	23.95	1	**< 0.001**	32.00	1	**< 0.001**
Habitat:sex	2526.47	1	**< 0.001**	1954.82	1	**< 0.001**

*Note:* Significant *p* values are highlighted in bold.

**FIGURE 7:**
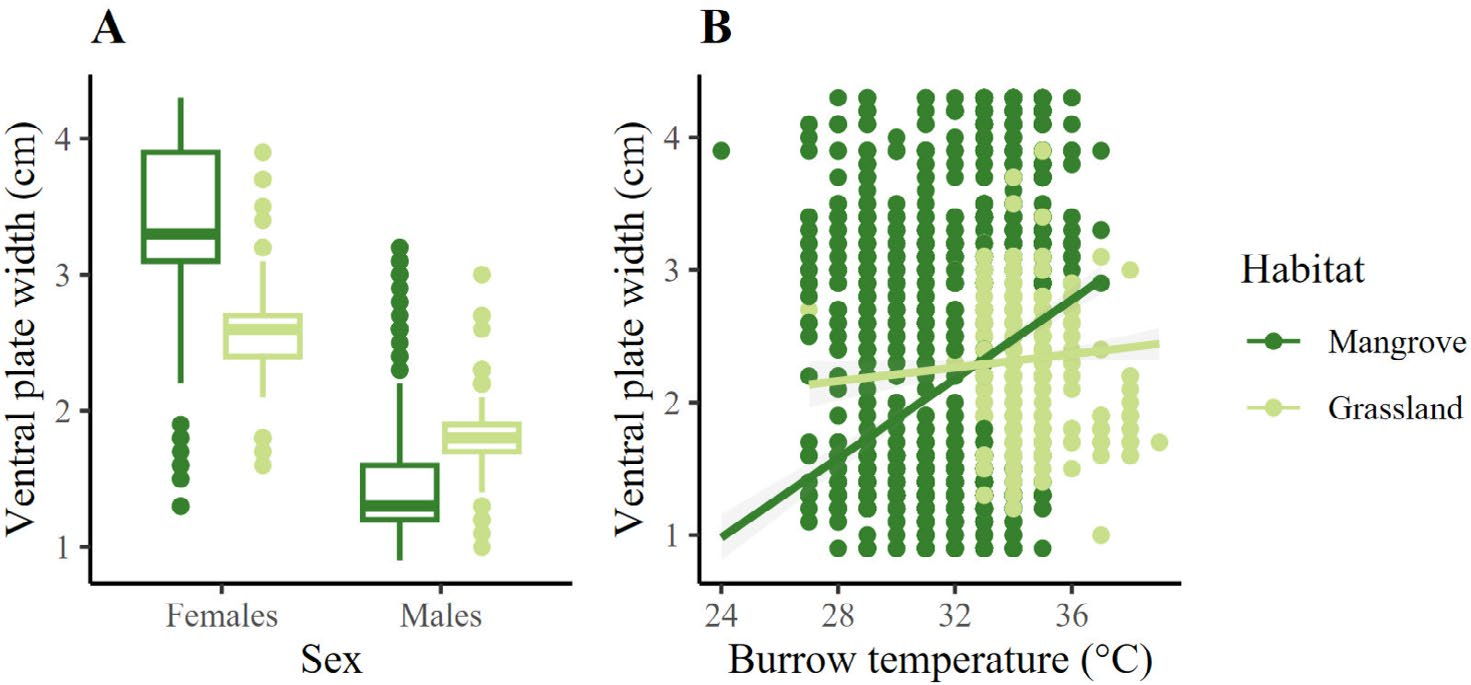
(A) Ventral plate size of females and males in contrasting habitats. Mean and extreme values are represented. (B) Relationship predicted by the LMM between ventral plate size and burrow temperature. Ventral plate size increased with burrow temperature in the mangrove.

Finally, habitat, sex, burrow temperature, and the interaction habitat:sex had a significant effect on ventral plate width (Table 3). The ventral plate of females was wider in the mangrove than in the pasture (Figure 7A; Table 3). There was also a positive relationship between ventral plate width and burrow temperature, although it was more evident in the mangrove than in the pasture (Figure 7B).

## 4 Discussion

We investigated the influence of the habitat and environmental conditions (environmental and burrow temperature and soil hardness) on the ASR, density, and physiological and morphological traits of *C. guanhumi* at a regional scale across 12 sampling sites on the coast of the Gulf of Mexico. Our results support our prediction that *C. guanhumi* populations in mangroves exhibit higher density, higher mean body condition (size, weight), and a biased ASR compared to those in pastures. To the best of our knowledge, this is the first study to show an effect of habitat on demographic parameters (ASR and density) and morphological attributes in *C. guanhumi* at both a local and regional scale across a large part of the coast of the Gulf of Mexico.

According to our prediction, we found that habitat was the only predictor of ASR in *C. guanhumi* , since environmental and burrow temperature, soil hardness, and density did not influence this parameter. These results indicate that the bias towards males in the mangrove and towards females in the pasture at a local (by site) and regional (on the coasts of Tamaulipas and Veracruz in the Gulf of Mexico) scale is not influenced by these abiotic factors but rather by land‐use change. However, there are still not enough studies to understand the causes of variation in ASR, especially across spatial scales (Xu et al. 2016).

Biases in the sex ratio and their variation may reflect adaptations related to changes in the spatio‐temporal distribution of individuals and the characteristics of the habitat (climatic conditions, resource availability, among others) (Dittmar et al. 2011; Székely et al. 2014; Jennions and Fromhage 2017; László et al. 2018; Barretto et al. 2022). *Cardisoma guanhumi* has adapted to occupy different continental habitats, mainly through access to the water table (Govender and Rodríguez‐Fourquet 2008; Rodríguez‐Fourquet and Sabat 2009; Mendes and Cruz 2017; Novais et al. 2021). Furthermore, the flexibility of this crab species in terms of new food sources is leading to shifts in its demographic processes at all scales. For example, a higher abundance in modified habitats, such as cities, compared to natural habitats is related to a constant availability of food resources such as dead animals and human feces, which appear to be consumed by this species (Riascos et al. 2024).

We found a higher abundance of crabs in mangroves, according to our prediction. Several studies have found that populations in pastures are under greater predation and fishing pressure. Predation is a natural process but, in pastures, it is caused by mammals introduced by humans, such as stray dogs and cats, crab‐eating raccoons ( *Procyon cancrivorus* ), and opossums (*Didelphis* sp.), among others (Schwamborn and Moraes‐Costa 2019). High predation pressure in pastures increases the likelihood of mortality, which is reflected in smaller populations compared to mangroves.

In the case of *C. guanhumi* , we highlight ambient temperature and soil hardness as factors influencing its abundance. For example, the density of individuals was inversely proportional to soil hardness in both mangroves and pastures. However, the loss of vegetation in the pastures causes an increase in ambient and soil temperature, which affects the abundance of crabs in this habitat. Moreover, the soil in the burrows was more compacted in the pastures than in the mangrove, which explains a higher density of individuals in the mangrove compared to the pastures. Burrow construction and maintenance are costly activities due to the time and energy invested (reviewed in Lombardo and Rojas 2022). Thus, a higher crab density in the mangrove may be related to a continued use of burrows to ensure reproduction and protection from predators. On the other hand, the high density of crabs in mangroves suggests that this habitat is their natural ecosystem locally and regionally and is of better quality than pastures. Although *C. guanhumi* appears to have adapted to pastures, this habitat remains less suitable for its conservation (Oliviera‐Neto et al. 2014), as populations in low‐quality habitats show reduced individual survival and reproduction rates, which result in low densities, and may therefore require immigration of individuals from high‐quality habitats (Lea et al. 2018), such as mangroves, to persist.

The spatial distribution of a species can influence population parameters such as ASR or density, especially in the case of species that can establish in different types of habitats (Arroyave‐Rincón et al. 2014). The analysis of density and ASR in each habitat at the spatial scales used in this study (local and regional) suggests (based on Gundersen et al. 2001) that *C. guanhumi* dispersal may depend on these two factors, which may, therefore, influence the source‐sink dynamics in this species. That is, females in pastures (sink habitats) are likely the sex with greater dispersal, which is reflected in a lower population density than that found in source habitats (mangroves), where they originate. Furthermore, based on our prediction, ASR was biased towards the sex with the greatest ability to exploit the environmental conditions of the habitat. Hence, ASR was biased towards males in mangroves, and thus the environmental conditions in this habitat seem to favor their lower dispersal, as has been found in other studies (Julliard 2000; Steifetten and Dale 2012; Végvári et al. 2018, Barretto et al. 2022).

The variation in the ASR along the regional distribution of this species in the Gulf of Mexico may have significant implications for its reproductive biology and, consequently, for the survival of the population. The bias towards sexually mature males observed in the mangrove in the present study has also been recorded in regions with weak predation pressure, primarily in populations restricted to islands (Hernández‐Maldonado and Campos Campos 2015). The relationship between geographic distribution, population growth, and population viability may be complex in *C. guanhumi* and depend on a range of characteristics, including density and mating system. For example, according to our results, a low number of males in the pasture at a low population density suggests reduced female fecundity as a result of the limited capacity of males to fertilize all the females (Emlen and Oring 1977; Shuster and Wade 2003). In the case of the bias towards males in the mangrove, a different approach predicts adaptive behavioral responses to a biased ASR. A male‐biased ASR in the mangrove may improve female fertility by increasing the chance of selecting a mate and, therefore, producing more viable offspring, as has been observed in other studies (Emlen and Oring 1977; Julliard 2000; Shuster and Wade 2003). It would be interesting to explore these hypotheses based on mating systems theory in future studies. It is important to analyze the causes of variation in the ASR to understand the relationship between ASR, mating system, and population dynamics in this species.

Temperature is an important factor in the establishment and development of *C. guanhumi* (Govender and Rodríguez‐Fourquet 2008; Carmona‐Suárez and Guerra‐Castro 2012; Giménez et al. 2015; Mendes and Cruz 2017). In the present study, we observed that environmental temperature influences the density of individuals. Furthermore, the temperature inside the burrow (where *C. guanhumi* spends most of its life and is always lower than the environmental temperature) has an effect on its development. Gómez‐Cervantes (2020) reported that temperature is one of the main factors contributing to the optimal development of this species. In our study, we found that, at least in the mangrove, temperature favored an increase in weight and carapace and ventral plate size. The quela was the only structure that was not influenced by temperature. The size of most morphological structures increased at a lower burrow temperature in the mangrove (27.5°C–32°C) compared to the pasture (32°C–38°C). This indicates that shifts towards a smaller body size are associated with the pastures. The size of most morphological structures increased at lower burrow temperatures in the mangrove. An increased weight and structure size may compensate for a lower burrow temperature in the mangrove, while heat stress in pastures may explain the more stable weight and size of the crabs in this habitat. Similar to other organisms, *C. guanhumi* responds to land‐use change associated with the transformation of mangroves into pastures by decreasing their body size. In these ecological conditions the populations of this crab species could possibly reach maturity at a smaller size, following the prediction of the “temperature‐size rule” presented in most ectotherms (Daufresne et al. 2009; Verberk et al. 2021).

The few studies that examine differences in the size of *C. guanhumi* between populations and habitats report a significantly larger size in mangrove forests compared to pastures in Colombia (Arroyave‐Rincón et al. 2014; Carmona‐Suárez and Guerra‐Castro 2018). However, size measurements were not obtained by sex, as in the present study. These results indicate habitat segregation by condition and size in both sexes of *C. guanhumi* . Differences in size by habitat and sex appear to be related to reproductive system and ecological differences between the sexes, as observed in some species (Kennedy and Rennie 2024). It has been found that, when there are more females (as is the case of mangroves), most of them invest less energy in gonad development, resulting in increased body growth (Silva et al. 2014), which could explain why females are larger in mangroves. This could have repercussions on mating systems. For example, laboratory experiments have shown that, under high‐quality conditions (greater availability of food and egg‐laying sites), males avoid mating with small females, while females are more selective when they are in low‐quality environments (Gillespie et al. 2014).

This crab species shows sexual dimorphism in ventral plate size, where it is wider in females than in males (Quiñones‐Llópiz et al. 2021). This pattern was observed in both mangroves and pastures. However, the ventral plate of females was narrower in pastures than in mangroves. This difference may have implications for the transportation of eggs, since females of this species undertake spawning migrations, traveling up to 12 km to release the larvae in a body of water (Giménez et al. 2015). In addition to providing better shelter than pastures, mangroves are located next to bodies of water. Therefore, spawning migrations are shorter in this habitat. The above suggests that females in mangroves carry a higher number of eggs, since they are protected by a wider ventral plate compared to females in pastures. On the other hand, the carapace has been considered an indicator of growth in this species, as in *Cardisoma crassum* (Molina‐Ortega and Vázquez‐López 2018). Similarly to the other structures, variation in carapace width in *C*. *guahumi* is related to adaptive strategies according to the habitat and burrow temperature. Other studies have shown that carapace width responds to habitat loss, as well as to local fishing pressure (Silva et al. 2014; Hernández‐Maldonado and Campos Campos 2015; Govender 2019).

A distinctive trait of *C. guanhumi* is the size of its quela, since they are the largest and most conspicuous part of the body of most individuals. Exaggerated morphological structures are often associated with sexual selection (Andersson 1994), but natural selection can also favor extreme morphologies that enhance foraging or locomotion (Bro‐Jørgensen 2008). The quela is a multifunctional structure in *C. guanhumi* , since it is used for feeding, fighting, and protection. The crabs also use their quela to dig burrows over 2 m deep, where they spend most of their lives (Mendes and Cruz 2017). Given the variety of functions the quela performs and the distribution of this species in contrasting habitats such as mangroves and pastures, morphological changes would be expected in this structure. There is evidence that the environment can limit the size of structures. For example, the cranial appendages of bovids and cervids are shaped by their environment, where species inhabiting open pastures exhibit larger structures and those inhabiting dense forests have smaller or absent structures (Caro et al. 2003; Cabrera and Stankowich 2020). In other crab species, such as *Cambarus chasmodactylus* , ecological factors like the vegetation composition and substrate influence their distribution and quela morphology depending on whether they are found in lotic or lentic sites in rivers or streams (Graham 2021). Our study shows that quela thickness in *C. guanhumi* varies with sex and habitat, where females in mangroves and males in pastures have thicker quela. Since the quela is a multifunctional structure, the larger quela size exhibited by males in pastures is likely related to territoriality involving resources and/or mates (Moraes‐Costa and Schwamborn 2018; Riascos et al. 2024). Accelerated quela growth in mature individuals is associated with territory guarding and male–male competition for females, as observed in most decapods (Graham 2021). Furthermore, sex‐specific behavioral differences, such as habitat choice and food preference, have been described in crabs such as *Armases cinereum* in coastal forests and salt marshes on Sapelo Island (Moraes‐Costa and Schwamborn 2018). According to this, there may be selection on the feeding behavior of *C. guanhumi* , where females with larger quelae in mangroves may be more efficient at foraging, perhaps allowing them to develop this structure more efficiently than smaller males in this habitat. This implies fitness benefits for females, as a larger body size is associated with greater offspring size and survival (Hübneṙ et al. 2015). Furthermore, the diet of *C. guanhumi* has also been found to differ between habitats, where it collects fallen leaves and fruits in mangroves, while it has been observed clipping the vegetation to feed on it in pastures (Herreid 1963). It is recommended to study the influence of the habitat on feeding behavior, mating strategy, and sexual dimorphism of morphological traits in *C. guanhumi* at a local and regional level through morphometric analyses and its relationship with the developmental patterns of the gonads.

## 5 Conclusion

The results of our study indicate that *C. guanhumi* is sensitive to the type of habitat. At a regional scale, in the coast of the Gulf of Mexico, *C. guanhumi* showed a preference for mangroves. This habitat had suitable conditions (burrow temperature and soil hardness) that favored a higher crab density and a male‐biased sex ratio. Furthermore, there were differences between sexes in morphological and physiological traits which, particularly in females, varied with the habitat and environmental conditions. These traits showed that females in mangroves were in better condition than those in pastures. The demographic and morphological responses to contrasting habitats show that this species has different requirements. This suggests that crabs in mangroves are more likely to survive and reproduce than those in human‐modified environments such as pastures. This type of study provides key information on the population ecology of this species for the implementation of conservation measures, since it may be threatened by commercial exploitation by persisting in modified environments such as pastures.

## Author Contributions

**Jared Leyva‐Hernández:** conceptualization (equal), data curation (equal), formal analysis (equal), methodology (equal). **Martha L. Baena:** conceptualization (equal), investigation (equal), project administration (equal), visualization (equal), writing – original draft (equal), writing – review and editing (equal). **Ivette Alicia Chamorro‐Florescano:** conceptualization (equal), investigation (equal), supervision (equal), validation (equal), visualization (equal), writing – review and editing (equal). **Israel Huesca‐Domínguez:** formal analysis (equal), methodology (equal), software (equal), supervision (equal), visualization (equal), writing – review and editing (equal).

## Ethics Statement

This study did not involve the killing of any individuals; they were all released into their respective burrows after collecting the data.

## Conflicts of Interest

The authors declare no conflicts of interest.

## Supporting information


Table S1.

Table S2.

## Data Availability

All data used in the study are included in this paper and are available.

## References

[ece371710-bib-0001] Alonso‐Fernández, A. , J. Otero , R. Bañón , J. M. Campelos , J. Santos , and G. Mucientes . 2017. “Sex Ratio Variation in an Exploited Population of Common Octopus: Ontogenic Shifts and Spatio‐Temporal Dynamics.” Hydrobiologia 794: 1–16. 10.1007/s10750-016-3065-3.

[ece371710-bib-0002] Amaral, M. R. X. , M. Albrecht , A. S. Mckinley , A. M. F. De Carvalho , S. Cavalcante‐De Sousa , and F. M. Diniz . 2015. “Mitochondrial DNA Variation Reveals a Sharp Genetic Break Within the Distribution of the Blue Land Crab *Cardisoma guanhumi* in the Western Central Atlantic.” Molecules 20: 15158–15174. 10.3390/molecules200815158. 26295384 PMC6332107

[ece371710-bib-0003] Amos, J. N. , S. Balasubramaniam , L. Grootendorst , et al. 2013. “Little Evidence That Condition, Stress Indicators, Sex Ratio, or Homozygosity Are Related to Landscape or Habitat Attributes in Declining Woodland Birds.” Journal of Avian Biology 44, no. 1: 45–54. 10.1111/j.1600-048X.2012.05746.x.

[ece371710-bib-0004] Ancona, S. , F. V. Dénes , O. Krüger , T. Székely , and S. R. Beissinger . 2017. “Estimating Adult Sex Ratios in Nature.” Philosophical Transactions of the Royal Society, B: Biological Sciences 372, no. 1729: 20160313. 10.1098/rstb.2016.0313. PMC5540855 28760756

[ece371710-bib-0005] Andersson, M. 1994. Sexual Selection. Princeton University Press.

[ece371710-bib-0006] Arroyave‐Rincón, A. , V. Amortegui‐Torres , J. F. Blanco‐Libreros , and A. Marín . 2014. “Edge Effect on Blue Crab Population *Cardisoma guanhumi* (Decapoda: Gecarcinidae) in the Mangrove of El Uno Bay, Uraba Gulf (Colombia): An Approximation to the Folk Catchery.” Actualidades Biológicas 36, no. 100: 47–57.

[ece371710-bib-0007] Barretto, J. W. , M. L. Baena , I. H. Domínguez , and F. Escobar . 2022. “Spatiotemporal Variation in the Adult Sex Ratio, Male Aggregation, and Movement of Two Tropical Cloud Forest Dung Beetles.” Current Zoology 68, no. 6: 635–644. 10.1093/cz/zoab101. 36743229 PMC9892795

[ece371710-bib-0008] Bates, D. , M. Mächler , B. Bolker , and S. Walker . 2015. “Fitting Linear Mixed‐Effects Models Using lme4.” Journal of Statistical Software 67, no. 1: 1–48. 10.18637/jss.v067.i01.

[ece371710-bib-0009] Bókony, V. , C. Kalina , N. Ujhegyi , et al. 2024. “Does Stress Make Males? An Experiment on the Role of Glucocorticoids in Anuran Sex Reversal.” Journal of Experimental Zoology Part A: Ecological and Integrative Physiology 341, no. 2: 172–181. 10.1002/jez.2772. 38155497

[ece371710-bib-0010] Bro‐Jørgensen, J. 2008. “Dense Habitats Selecting for Small Body Size: A Comparative Study on Bovids.” Oikos 117, no. 5: 729–737. 10.1111/j.0030-1299.2008.16069.x.

[ece371710-bib-0011] Burgos, G. P. , and C. A. Garófalo . 2018. “Afectación del cambio climático en la captura y comercialización del cangrejo azul (*Cardosma guanhumi*) en el Ecuador.” DELOS: Desarrollo Local Sostenible 11, no. 31: 1–10.

[ece371710-bib-0012] Cabrera, D. , and T. Stankowich . 2020. “Stabbing Slinkers: Tusk Evolution Among Artiodactyls.” Journal of Mammalian Evolution 27, no. 2: 265–272. 10.1007/s10914-018-9453-x.

[ece371710-bib-0013] Canty, A. , and B. Ripley . 2017. “Package ‘boot’. Bootstrap Functions.” CRAN R Project.

[ece371710-bib-0014] Capistrán‐Barradas, A. , O. Defeo , and P. Moreno‐Casasola . 2003. “Density and Population Structure of the Red Land Crab *Gecarcinus lateralis* in a Tropical Semi‐Deciduous Forest in Veracruz, Mexico.” Interciencia 28, no. 6: 323–327.

[ece371710-bib-0015] Carmona‐Isunza, M. C. , S. Ancona , T. Székely , et al. 2017. “Adult Sex Ratio and Operational Sex Ratio Exhibit Different Temporal Dynamics in the Wild.” Behavioral Ecology 28: 523–532. 10.1093/beheco/arw183.

[ece371710-bib-0016] Carmona‐Suárez, C. A. , and E. Guerra‐Castro . 2012. “Comparison of Three Quick Methods to Estimate Crab Size in the Land Crabs *Cardisoma guanhumi* Latreille, 1825 and *Ucides cordatus* (Crustacea: Brachyura: Gecarcinidae and Ucididae).” Revista de Biología Tropical 60: 139–149.

[ece371710-bib-0017] Carmona‐Suárez, C. A. , and E. J. Guerra‐Castro . 2018. “Populations of *Cardisoma guanhumi* Latreille in Latreille, Le Peletier, Serville & Guérin, 1828 (Decapoda: Brachyura: Gecarcinidae) in Mangrove Forests and Coastal Grasslands in Venezuela.” Journal of Crustacean Biology 38, no. 6: 739–747. 10.1093/jcbiol/ruy074.

[ece371710-bib-0018] Caro, T. M. , C. M. Graham , C. J. Stoner , and M. M. Flores . 2003. “Correlates of Horn and Antler Shape in Bovids and Cervids.” Behavioral Ecology and Sociobiology 55: 32–41. 10.1007/s00265-003-0672-6.

[ece371710-bib-0019] Clutton‐Brock, T. H. 1991. “The Evolution of Sex Differences and the Consequences of Polygyny in Mammals.” In The Development and Integration of Behaviour: Essays in Honour of Robert Hinde, 229–253. Cambridge University Press.

[ece371710-bib-0082] Daufresne, M. , K. Lengfellner , and U. Sommer . 2009. “Global Warming Benefits the Small in Aquatic Ecosystems.” Proceedings of the National Academy of Sciences of the United States of America 106, no. 31: 12788–12793. 19620720 10.1073/pnas.0902080106 PMC2722360

[ece371710-bib-0020] Davison, A. C. , and D. V. Hinkley . 1997. Bootstrap Methods and Their Applications. Cambridge University Press.

[ece371710-bib-0021] Dittmar, K. , S. Morse , M. Gruwell , J. Mayberry , and E. DiBlasi . 2011. “Spatial and Temporal Complexities of Reproductive Behavior and Sex Ratios: A Case From Parasitic Insects.” PLoS One 6, no. 5: e19438. 10.1371/journal.pone.0019438. 21572996 PMC3091855

[ece371710-bib-0022] Edmands, S. 2021. “Sex Ratios in a Warming World: Thermal Effects on Sex‐Biased Survival, Sex Determination, and Sex Reversal.” Journal of Heredity 112, no. 2: 155–164. 10.1093/jhered/esab006. 33585893

[ece371710-bib-0023] Emlen, S. T. , and L. W. Oring . 1977. “Ecology, Sexual Selection, and the Evolution of Mating Systems.” Science 197, no. 4300: 215–223. 10.1126/science.327542. 327542

[ece371710-bib-0024] Fisher, R. A. 1930. “Sexual Reproduction and Sexual Selection.” In The Genetical Theory of Natural Selection. Clarendon Press.

[ece371710-bib-0025] Fromhage, L. , and M. D. Jennions . 2016. “Coevolution of Parental Investment and Sexually Selected Traits Drives Sex‐Role Divergence.” Nature Communications 7, no. 1: 12517. 10.1038/ncomms12517. PMC4992156 27535478

[ece371710-bib-0026] Gifford, C. A. 1962. “Some Observations on the General Biology of the Land Crab, *Cardisoma guanhumi* (Latreille), in South Florida.” Biological Bulletin 123, no. 1: 207–223.

[ece371710-bib-0027] Gillespie, S. R. , M. Scarlett Tudor , A. J. Moore , and C. W. Miller . 2014. “Sexual Selection Is Influenced by Both Developmental and Adult Environments.” Evolution 68, no. 12: 3421–3432. 25226860 10.1111/evo.12526

[ece371710-bib-0028] Giménez, E. , Y. Garcés , Y. González , and A. Hurtado . 2015. “Densidad poblacional de *Cardisoma guanhumi* (Latreille, 1825) (Crusácea: Gercarcinidae) en el Parque Nacional Ciénaga de Zapata, Cuba.” Boletín Centro de Investigación Biológica 49, no. 2: 110–124.

[ece371710-bib-0029] Gómez‐Cervantes, S. K. 2020. “Desarrollo del ciclo larval del Cangrejo Azul *Cardisoma guanhumi* Latreille, 1828 en sistemas cerrados.” Doctoral dissertation, Universidad del Sinú, seccional Cartagena.

[ece371710-bib-0030] Govender, Y. 2019. “Long‐Term Monitoring of Crab *Cardisoma guanhumi* (Decapoda: Gecarcinidae) Captures in Jobos Bay Estuary, Puerto Rico.” Revista de Biología Tropical 67, no. 4: 879–887. 10.15517/rbt.v67i4.35124.

[ece371710-bib-0031] Govender, Y. , and C. Rodríguez‐Fourquet . 2008. “Techniques for Rapid Assessment of Population Density and Body Size of the Land Crab *Cardisoma guanhumi* (Lattreille, 1825) in Puerto Rico.” Tropical Estuaries 1: 9–15.

[ece371710-bib-0032] Graham, Z. A. 2021. “Moving in Fast Waters: The Exaggerated Claw Gape of the New River Crayfish (*Cambarus chasmodactlyus*) Aids in Locomotor Performance.” Biology Letters 17, no. 5: 20210045. 10.1098/rsbl.2021.0045. 34006118 PMC8131936

[ece371710-bib-0079] Gundersen, G. , E. Johannesen , H. P. Andreassen , and R. A. Ims . 2001. “Source–sink Dynamics: How Sinks Affect Demography of Sources.” Ecology Letters 4, no. 1: 14–21.

[ece371710-bib-0033] Hardy, I. C. V. 2002. Sex Ratios. Cambridge University Press.

[ece371710-bib-0034] Hartnoll, R. G. , C. Régnier‐McKellar , N. Weber , and S. B. Weber . 2014. “Return to the Land; the Stages of Terrestrial Recruitment in Land Crabs.” Crustaceana 87, no. 5: 531–539.

[ece371710-bib-0035] Hernández‐Maldonado, A. , and N. H. Campos Campos . 2015. “Estado actual de la población adulta del cangrejo semiterrestre *Cardisoma guanhumi* (Latreille) en la isla de San Andrés, Caribe Colombiano.” Boletín de Investigaciones Marinas y Costeras‐INVEMAR 44, no. 1: 185–198.

[ece371710-bib-0036] Hernández‐Melchor, G. I. , O. Ruíz Rosado , A. Sol Sánchez , and J. I. Valdez Hernández . 2016. “Cambios de uso del suelo en manglares de la costa de Tabasco.” Revista Mexicana de Ciencias Agrícolas 7, no. spe14: 2757–2767.

[ece371710-bib-0037] Herreid, C. F. 1963. “Observations on the Feeding Behavior of *Cardisoma guanhumi* (Latreille) in Southern Florida.” Crustaceana 5: 176–180.

[ece371710-bib-0038] Hübneṙ, L. , S. C. Pennings , and M. Zimmer . 2015. “Sex‐and Habitat‐Specific Movement of an Omnivorous Semi‐Terrestrial Crab Controls Habitat Connectivity and Subsidies: A Multi‐Parameter Approach.” Oecologia 178: 999–1015. 25783486 10.1007/s00442-015-3271-0

[ece371710-bib-0039] Jennions, M. D. , and L. Fromhage . 2017. “Not All Sex Ratios Are Equal: The Fisher Condition, Parental Care and Sexual Selection.” Philosophical Transactions of the Royal Society, B: Biological Sciences 372, no. 1729: 20160312. 10.1098/rstb.2016.0312 PMC5540854 28760755

[ece371710-bib-0041] Julliard, R. 2000. “Sex‐Specific Dispersal in Spatially Varying Environments Leads to Habitat‐Dependent Evolutionary Stable Offspring Sex Ratios.” Behavioral Ecology 11: 421–428.

[ece371710-bib-0042] Kappeler, P. M. , S. Benhaiem , C. Fichtel , et al. 2023. “Sex Roles and Sex Ratios in Animals.” Biological Reviews 98, no. 2: 462–480. 36307924 10.1111/brv.12915

[ece371710-bib-0043] Kennedy, P. J. , and M. D. Rennie . 2024. “Variation in Female‐Biased Sexual Size Dimorphism of Northern Pike (*Esox lucius*) Associated With Environment and Life History.” Evolutionary Ecology 38: 1–22.

[ece371710-bib-0044] Kokko, H. , and D. J. Rankin . 2006. “Lonely Hearts or Sex in the City? Density‐Dependent Effects in Mating Systems.” Philosophical Transactions of the Royal Society, B: Biological Sciences 361, no. 1466: 319–334. 10.1098/rstb.2005.1784 PMC1569612 16612890

[ece371710-bib-0078] Lea, J. M. , S. L. Walker , G. I. Kerley , J. Jackson , S. C. Matevich , and S. Shultz . 2018. “Non‐invasive Physiological Markers Demonstrate Link Between Habitat Quality, Adult Sex Ratio and Poor Population Growth Rate in a Vulnerable Species, the Cape Mountain Zebra.” Functional Ecology 32, no. 2: 300–312.

[ece371710-bib-0045] László, Z. , A. L. Dénes , L. Király , and B. Tóthmérész . 2018. “Biased Parasitoid Sex Ratios: *Wolbachia*, Functional Traits, Local and Landscape Effects.” Basic and Applied Ecology 31: 61–71.

[ece371710-bib-0047] Lombardo, R. C. , and M. Rojas . 2022. “Burrow Fidelity in the Blue Crab, *Cardisoma crassum* Smith, 1870 (Brachyura: Gecarcinidae) From the Ponuga River, Veraguas, Panama.” Nauplius 30: e2022033.

[ece371710-bib-0075] Marrero, H. J. , C. Gómez‐Martínez , M. L. Allasino , J. P. Haedo , M. A. González‐Estévez , and A. Lázaro . 2025. “Local and Landscape Effects on the Reproduction of Wild Bees and Wasps in Mediterranean Communities Along a Gradient of Land‐Use.” Ecological Entomology 50, no. 2: 1–12.

[ece371710-bib-0048] Mendes, L. D. N. , and R. Cruz . 2017. “Estimation of Density and Abundance of the Blue Land Crab, *Cardisoma guanhumi* Latreille, 1828, in the Imburana Peninsula, Northern Brazil.” Crustaceana 90, no. 5: 571–587.

[ece371710-bib-0049] Molina‐Ortega, M. G. , and H. Vázquez‐López . 2018. “Crecimiento relativo de *Cardisoma crassum* smith, 1870 (Decapoda: Gecarcinidae) en el estero el Salado, Puerto Vallarta, Jalisco México.” Biología, Ciencia y Tecnología BIOCYT 11, no. 43: 808–823.

[ece371710-bib-0050] Moraes‐Costa, D. , and R. Schwamborn . 2018. “Site Fidelity and Population Structure of Blue Land Crabs (*Cardisoma guanhumi* Latreille, 1825) in a Restricted‐Access Mangrove Area, Analyzed Using PIT Tags.” Helgoland Marine Research 72: 1–15.

[ece371710-bib-0051] Mota, T. A. , M. A. A. Pinheiro , N. S. Evangelista‐Barreto , and S. S. da Rocha . 2023. “Density and Extractive Potential of “Uçá” Crab, *Ucides cordatus* (Linnaeus, 1763), in Mangroves of the “Todos Os Santos” Bay, Bahia, Brazil.” Fisheries Research 26: 106733.

[ece371710-bib-0052] Novais, W. R. , F. L. Carvalho , and E. C. Couto . 2021. “Conservation of the Endangered Blue Land Crab *Cardisoma guanhumi* Latreille in Latreille, Le Peletier, Serville & Guérin, 1828 (Decapoda: Brachyura: Gecarcinidae) in Brazil: Optimal Habitats and Environmental Factors.” Journal of Crustacean Biology 41, no. 2: ruab011.

[ece371710-bib-0077] Oliveira‐Neto, J. F. , E. Batista , R. Metri , and C. B. Metri . 2014. “Local Distribution and Abundance of *Cardisoma guanhumi* Latreille, 1928 (Brachyura: Gecarcinidae) in Southern Brazil.” Brazilian Journal of Biology 74, no. 1: 1–07. 10.1590/1519-6984.02912 25075466

[ece371710-bib-0053] Osland, M. J. , A. R. Hughes , A. R. Armitage , et al. 2022. “The Impacts of Mangrove Range Expansion on Wetland Ecosystem Services in the Southeastern United States: Current Understanding, Knowledge Gaps, and Emerging Research Needs.” Global Change Biology 28, no. 10: 3163–3187. 35100489 10.1111/gcb.16111

[ece371710-bib-0054] Portillo, J. L. , and E. Ezcurra . 2002. “Los manglares de México: una revisión.” Madera y Bosques 8: 27–51.

[ece371710-bib-0055] Quiñones‐Llópiz, J. D. , C. Rodríguez‐Fourquet , T. Luppi , and N. E. Farias . 2021. “Size Distribution and Sex Ratio Between Populations of the Artisanal Harvested Land Crab *Cardisoma guanhumi* (Decapoda: Gecarcinidae), With the Estimation of Relative Growth and Size at Sexual Maturity in Puerto Rico.” Revista de Biología Tropical 69, no. 3: 989–1003.

[ece371710-bib-0056] R Core Team . 2024. R: A Language and Environment for Statistical Computing. R Foundation for Statistical Computing.

[ece371710-bib-0057] Ramos, J. , M. Boto , J. F. Blanco‐Libreros , and J. M. Riascos . 2021. “The Mangrove Periwinkle *Littoraria angulifera* (Mollusca: Littorinidae) in the Urabá Gulf (Colombian Caribbean): Finding Ways in an Urbanizing Coast?” Frontiers in Marine Science 8: 641567.

[ece371710-bib-0058] Reichard, M. , M. Polačik , R. Blažek , and M. Vrtílek . 2014. “Female Bias in the Adult Sex Ratio of African Annual Fishes: Interspecific Differences, Seasonal Trends and Environmental Predictors.” Evolutionary Ecology 28: 1105–1120.

[ece371710-bib-0059] Riascos, J. M. , L. D. Obonaga , and J. Ramos . 2024. “Is the Threatened Land Crab *Cardisoma guanhumi* Conquering Human‐Dominated Systems?” Ecology and Evolution 14, no. 4: e10737. 38681183 10.1002/ece3.10737 PMC11046080

[ece371710-bib-0060] Rodríguez‐Fourquet, C. , and A. M. Sabat . 2009. “Effect of Harvesting, Vegetation Structure and Composition on the Abundance and Demography of the Land Crab *Cardisoma guanhumi* in Puerto Rico.” Wetlands Ecology and Management 17: 627–640.

[ece371710-bib-0061] Rohde, K. 1992. “Latitudinal Gradients in Species Diversity: The Search for the Primary Cause.” Oikos 65: 514–527. 10.2307/3545569.

[ece371710-bib-0062] Santos, M. C. F. , E. R. O. Botelho , I. H. A. Cintra , A. V. Barreto , K. C. A. Silva , and J. O. Branco . 2016. “Caracterização topográfica do habitat do *Cardisoma guanhumi* Latreille, 1828 (Decapoda, Gecarcinidae) na Apa Costa dos Corais (Pernambuco e Alagoas, Brasil).” Biota Amazônia 6, no. 3: 102–107.

[ece371710-bib-0063] Schacht, R. , S. R. Beissinger , C. Wedekind , et al. 2022. “Adult Sex Ratios: Causes of Variation and Implications for Animal and Human Societies.” Communications Biology 5, no. 1: 1273. 36402823 10.1038/s42003-022-04223-w PMC9675760

[ece371710-bib-0064] Schwamborn, R. , and D. F. Moraes‐Costa . 2019. “Growth and Mortality of Endangered Land Crabs (*Cardisoma guanhumi*) Assessed Through Tagging With PITs and Novel Bootstrapped Methods.” Preprint, arXiv. arXiv:1909.03311. 10.48550/arXiv.1909.03311.

[ece371710-bib-0065] Shuster, S. M. , and M. J. Wade . 2003. Mating Systems and Strategies. Princeton University Press.

[ece371710-bib-0066] Silva, C. C. , R. C. Schwamborn , and J. L. Oliveira . 2014. “Population Biology and Color Patterns of the Blue Land Crab, *Cardisoma guanhumi* (Latreille 1828) (Crustacea: Gecarcinidae) in the Northeastern Brazil.” Brazilian Journal of Biology 74, no. 4: 949–958. 10.1590/1519-6984.01913 25627608

[ece371710-bib-0067] Steifetten, Ø. , and S. Dale . 2012. “Dispersal of Male Ortolan Buntings Away From Areas With Low Female Density and a Severely Male‐Biased Sex Ratio.” Oecologia 168: 53–60. 10.1007/s00442-011-2082-1. 21800058

[ece371710-bib-0068] Székely, T. , F. J. Weissing , and J. Komdeur . 2014. “Adult Sex Ratio Variation: Implications for Breeding System Evolution.” Journal of Evolutionary Biology 27, no. 8: 1500–1512. 24848871 10.1111/jeb.12415

[ece371710-bib-0069] Végvári, Z. , G. Katona , B. Vági , et al. 2018. “Sex‐Biased Breeding Dispersal Is Predicted by Social Environment in Birds.” Ecology and Evolution 8, no. 13: 6483–6491. 10.1002/ece3.4095. 30038750 PMC6053579

[ece371710-bib-0070] Verberk, W. C. , D. Atkinson , K. N. Hoefnagel , A. G. Hirst , C. R. Horne , and H. Siepel . 2021. “Shrinking Body Sizes in Response to Warming: Explanations for the Temperature–Size Rule With Special Emphasis on the Role of Oxygen.” Biological Reviews 96, no. 1: 247–268. 32959989 10.1111/brv.12653 PMC7821163

[ece371710-bib-0071] Verma, S. , D. Sagar , H. Kumar , and G. S. Sujatha . 2024. “Abiotic Factors as Game Changer in Sex Ratio Distortion of Insects.” International Journal of Environment and Climate Change 14, no. 7: 332–342. 10.9734/ijecc/2024/v14i74274.

[ece371710-bib-0072] Wittmann, K. , A. M. Klein , and M. Staab . 2023. “The Influence of Habitat Properties on Sex Determination in Cavity‐Nesting Hymenoptera.” Basic and Applied Ecology 70: 1–11. 10.1016/j.baae.2023.04.001.

[ece371710-bib-0073] Xu, M. , M. Fang , Y. Yang , et al. 2016. “Spatial Variation in Adult Sex Ratio Across Multiple Scales in the Invasive Golden Apple Snail, *Pomacea canaliculata* .” Ecology and Evolution 6, no. 8: 2308–2317. 10.1002/ece3.2043. 27069581 PMC4782258

[ece371710-bib-0074] Yates, M. L. , N. R. Andrew , M. Binns , and H. Gibb . 2014. “Morphological Traits: Predictable Responses to Macrohabitats Across a 300 km Scale.” PeerJ 2: e271. 10.7717/peerj.271. 24688850 PMC3961160

