# Supplementary material for: Variation in the Adult Sex Ratio and Morphological Traits of Cardisoma guanhumi (Latreille, 1828) in Contrasting Habitats in the Southwest of the Gulf of Mexico
Source: Ecol Evol. 2025 Jul 9;15(7):e71710. doi: 10.1002/ece3.71710 (PMC12238770; doi:10.1002/ece3.71710)
Supplement: Supplementary file 1 — Table S1. [file ECE3-15-e71710-s002.docx]

**Table 1 *SM*.** Loadings of the principal component analysis (PCA) of the morphological variables of *Cardisoma guanhumi*.

| **Variables** | **Coordinates** | | **Contributions** | | | |  |
| --- | --- | --- | --- | --- | --- | --- | --- |
|  | **Dimension 1** | **Dimension 2** | | **Dimension 1** | | **Dimension 2** | |
| Carapace width | 0.95 | 0.10 | | 18.33 | | 1.65 | |
| Carapace length | 0.95 | 0.08 | | 18.37 | | 1.18 | |
| Chela width | 0.93 | -0.26 | | 17.59 | | 11.01 | |
| Chela length | 0.97 | -0.17 | | 18.81 | | 4.61 | |
| Chela thickness | 0.90 | -0.27 | | 16.21 | | 12.32 | |
| Ventral plate width | 0.73 | 0.65 | | 10.69 | | 69.22 | |
| Eigenvalues | 4.96 | 0.61 | |  |  | | |
| Explained variance | 82.73 | 10.15 | |  |  | | |
