# Supplementary material for: Variation in the Adult Sex Ratio and Morphological Traits of Cardisoma guanhumi (Latreille, 1828) in Contrasting Habitats in the Southwest of the Gulf of Mexico
Source: Ecol Evol. 2025 Jul 9;15(7):e71710. doi: 10.1002/ece3.71710 (PMC12238770; doi:10.1002/ece3.71710)
Supplement: Supplementary file 2 — Table S2. [file ECE3-15-e71710-s001.docx]

**Table 2 *SM*.** Morphological traits of *Cardisoma guanhumi* by habitat and sex on the coasts of Tamaulipas and Veracruz (Gulf of Mexico).

|  | **Females** | | **Males** | |  |  |
| --- | --- | --- | --- | --- | --- | --- |
| **Trait** | **Mangrove** | **Grassland** | **Grassland** | **Mangrove** | **χ²*** | **P** |
|  | **Mean±SD** | **Mean±SD** | **Mean±SD** | **Mean±SD** |  |  |
| Quela thickness | 1.5 (0.52) | 1.14 (0.35) | 1.48 (0.38) | 1.05 (0.41) | 792 | < .001 |
| Carapace width | 7.06 (1.01) | 5.5 (0.69) | 6.93 (0.81) | 5.28 (0.8) | 1885 | < .001 |
| Ventral plate width | 3.38 (0.52) | 2.61 (0.24) | 1.8 (0.24) | 1.38 (0.28) | 3038 | < .001 |
| *Kruskal-Wallis test | |  |  |  |  |  |
